# The Evaluation of Menstrual Alterations in Mexican Women After Vaccination Against COVID-19

**DOI:** 10.7759/cureus.58783

**Published:** 2024-04-22

**Authors:** Alejandra Contreras-Rendon, Edna Elisa Garcia Vences, Andrea Alicia Olguin-Ortega

**Affiliations:** 1 Department of Obstetrics and Gynecology, American British Cowdray (ABC) Medical Center, Mexico City, MEX; 2 Faculty of Health Sciences, Anahuac University, Mexico City, MEX; 3 Department of Gynecology, National Perinatology Institute, Mexico City, MEX

**Keywords:** uterine bleeding, multiple vaccine schedules, intermenstrual bleeding, covid-19 vaccination, menstrual disorders

## Abstract

Introduction

Menstrual changes after COVID-19 vaccination suggest a secondary connection to the immune response to vaccination rather than a specific component of the vaccine. The evaluation of these alterations in women with the same and multiple vaccination schedules will provide valuable information.

Methods

An observational, cross-sectional study was carried out; data were collected through a survey of 164 vaccinated women at the American British Cowdray (ABC) Santa Fe Medical Center Hospital in Mexico City. The survey was validated by the Delphi method.

Results

The survey was applied from March 2023 to February 2024. Post-vaccination menstrual alterations occurred in 48.1%; the most frequent alteration was menorrhagia in 20.7% and pain accompanied by menstruation in 27.4%. Fifty-seven percent had a history of previous COVID-19 infection. There were no significant associations between changes in menstrual bleeding after vaccination, history of COVID-19 infection, and age group (p>0.9). However, women who received multiple doses of vaccines had a higher risk of suffering abnormalities in bleeding by 36.6%.

Conclusion

The incidence of menstrual disorders in this study post COVID-19 vaccination was 49%. Menstrual alterations in patients who received multiple doses and a single regimen were similar at 47% and 48%, where there is no statistical significance. The greatest number of menstrual alterations was seen in the first dose at 36%, probably due to the immunity they acquired after the different types of vaccination. Vaccination is a very effective way to prevent the severity of COVID-19 infection; it has an impact on menstrual bleeding in terms of menorrhagia and metrorrhagia. Vaccination against COVID-19 is associated with small changes in the menstrual cycle, without statistical significance. Women receiving the first dose of the vaccine had changes in the amount of bleeding specifically the amount.

## Introduction

The onset of the COVID-19 pandemic has marked a global milestone, triggering significant advancements in science and medicine with the rapid development of various vaccines to mitigate the risks associated with the virus infection. Originating in late 2019 in China, the SARS-CoV-2 virus has sparked an unprecedented scientific race in the pursuit of treatments and vaccines to combat its spread [1].

Since then, international research laboratories and pharmaceutical companies have focused their efforts on developing vaccines to combat this disease. With notably high efficacy rates, current vaccines have played a crucial role in reducing the severe consequences of this infection. Among these, the Pfizer vaccine stands out, marking the beginning with efficacy in reducing the serious consequences of this COVID-19 infection of 95%, followed by AstraZeneca with 76% and Sputnik with 80% [1,2]. The incidence of menstrual alterations ranges from 24% to 40% depending on the country, and given its high incidence, it is of great interest to understand the behavior in our country [3].

All these vaccines have been widely distributed and have significantly contributed to the fight against the pandemic [4-7].

Before the onset of this health crisis, research on post-vaccination menstrual disturbances primarily focused on vaccines such as the human papillomavirus (HPV) or hepatitis B, with observations of heavier and more painful menstruations [5]. However, with the advent of COVID-19 vaccination, renewed attention has been paid to potential effects on the menstrual cycle [5,6].

The first indications of potential changes in the menstrual cycle following vaccination primarily emerged through social media platforms. Since June 2021, numerous reports of menstrual disorders have been received through various surveillance agencies, such as the Norwegian Medicines Agency and the UK Medicines and Healthcare products Regulatory Agency (MHRA). Additionally, the US Vaccine Adverse Event Reporting System (VAERS) has documented similar reports [8].

Despite these findings, clinical trials on adverse reactions to COVID-19 vaccines do not provide specific data on menstrual cycles post vaccination, and drug and vaccine adverse effects surveillance systems do not actively consider it either. This complicates the determination of a possible association between vaccination and menstrual disturbances [9].

The objective of this research was to assess the association of menstrual alterations following COVID-19 vaccination in Mexican women from the American British Cowdray (ABC) Medical Center who received vaccination with the same vaccination scheme and multiple vaccination schemes and, additionally, to evaluate if there was intermenstrual bleeding after vaccination.

## Materials and methods

Study design, setting, and participants

An observational, cross-sectional, survey-type study was carried out through Google Forms (Google, Inc., Mountain View, CA) from March 2023 to February 2024. The survey was carried out remotely by Dr. Alejandra Contreras. An email was used to send the invitation to the patients. In the same way, the main researcher can be contacted via email or telephone in case of doubt since the participants will be the patients of the office. The survey that was used was validated by experts before its application.

A questionnaire of 48 multiple-choice questions was carried out to be answered about the type of COVID-19 vaccine administered, number of doses, date of dose, multidoses, menstrual cycle pattern in women, number of days of bleeding, amount of bleeding, periods of menstruation, use of hormonal drugs, post-vaccination side effects, and previous COVID-19 condition. The validation process was in two phases; the first was sent to five doctors from different specialties for evaluation in terms of writing and understanding of the questions and answers. Subsequently, the second phase was sent to three gynecologists for validation. A pilot study was conducted with 10 women before distributing the questionnaire ensuring that all content was clear. This was validated using the Delphi method [10].

Tool Validation

A total of 164 women vaccinated against COVID-19 were evaluated in the gynecology outpatient clinic of the ABC Santa Fe Medical Center through a survey conducted remotely in Google Forms by Dr. Alejandra Contreras; they were given informed consent for authorization.

The data collected were analyzed using the Statistical Package for Social Sciences (SPSS) version 23.0 (IBM SPSS Statistics, Armonk, NY). A p-value of less than 0.05 was considered statistically significant. The sample size was calculated with the Epi Info program (Centers for Disease Control and Prevention, Atlanta, GA) for survey studies with a population size of 1,000, an expected frequency of 15%, and a 95% confidence interval, resulting in 164 patients.

A descriptive analysis was carried out to determine the clinical and demographic characteristics of the study participants, performing mental tests corroborated with the Kolmogorov-Smirnov test to determine normality, being considered normal when the significance of the test was greater than 0.05. For quantitative variables with normal distribution, the mean and standard deviation were used; for quantitative variables with free distribution, the median and interquartile range were used; and for nominal or ordinal qualitative variables, frequencies and proportions were used as descriptive statistics. Student's t-test was used for quantitative variables with normal distribution and the Mann-Whitney U test for quantitative variables with free distribution, and for qualitative variables, the Pearson χ^2^ test was used, but if in any cell the expected value was less than 5, Fisher's exact test was used.

## Results

A total of 164 women vaccinated against COVID-19 were studied, of which 39% were between 30 and 39 years old. More than half of the women (53%) are professionals, and only 17.1% are students. The majority of the participants had a history of previous COVID-19 infection, representing 57.3%. Of the women surveyed, all received the first dose of vaccination, and only 22% received the fourth dose. The most common type of COVID-19 vaccine for the first and second applications was Pfizer, with 46% and 43%, respectively (Table 1).

**Table 1:** Sociodemographic characteristics (n=164)

Variables		N (%)
Age in years	18-20	15 (9.1)
	21-29	52 (31.7)
	30-39	64 (39)
	40-49	26 (15.9)
	50-60	7 (4.3)
Occupation	Professional	87 (53)
	Homemaker	17 (10.4)
	Employee	27 (16.5)
	Student	28 (17.1)
	Others	5 (3)
Previous COVID-19 infection	Yes	94 (57.3)
	No	36 (22)
	I am not sure	34 (20)
Type of vaccine 1 doses	AstraZeneca	23 (14)
	Pfizer	76 (46.3)
	Moderna	22 (13.4)
	CanSino	3 (1.8)
	Johnson	17 (10.3)
	Sputnik	12 (7.3)
	Covaxin	0 (0)
	Sinovac	11 (6.7)
Type of vaccine 2 doses	AstraZeneca	31 (18.9)
	Pfizer	77 (46.9)
	Moderna	24 (14.6)
	CanSino	1 (0.6)
	Johnson	8 (4.8)
	Sputnik	13 (7.9)
	Covaxin	0 (0)
	Sinovac	10 (6)
Type of vaccine 3 doses	AstraZeneca	41 (25)
	Pfizer	38 (23.1)
	Moderna	23 (14)
	CanSino	3 (14)
	Johnson	3 (1.8)
	Sputnik	16 (9.7)
	Covaxin	0 (0)
	Sinovac	1 (0.6)
	Bivalent Pfizer	3 (1.8)
	None	36 (21.9)
Type of vaccine 4 doses	AstraZeneca	6 (3.6)
	Pfizer	9 (5.4)
	Moderna	14 (8.5)
	CanSino	1 (0.6)
	Johnson	1 (0.6)
	Sputnik	2 (1.2)
	Covaxin	0 (0)
	Sinovac	1 (0.6)
	Bivalent Pfizer	3 (1.8)
	None	127 (77.4)

Menstrual alterations were observed in 79 women with the same or multidose vaccine regimen, which represents 48.1% post vaccination. The most frequent alteration was an increase in the amount of bleeding in the menstrual cycle in 34 women (20.7%), while 9.7% reported a decrease in bleeding.

There were no significant associations between changes in post-vaccination menstrual bleeding, history of COVID-19 infection, and age group (p>0.9). However, women who received multiple doses of vaccine had a 75.9% higher risk of experiencing menstrual disorders than those who applied the same vaccination schedule (Table 2).

**Table 2:** Factors related to post-vaccination menstrual alterations

Variables		Menstrual alteration post vaccination		P-value
		Yes, 79 (48.12%)	No, 85 (51.8%)	
Type of vaccine received	Same type of vaccine	19 (24)	21 (24.7)	0.922
	Multiple types of vaccine	60 (75.9)	64 (75.2)	
	2 doses	36 (21.9)	128 (78)	0.12
	3 doses	18 (11)	110 (67)	
	4 doses	2 (1.2)	35 (21.3)	
Previous COVID-19 infection	Yes	42 (53.1)	52 (61.1)	0.555
	No	17 (21.5)	19 (22.3)	
	I am not sure	20 (25.3)	14 (16.4)	
Age in groups (years)	18-20	8 (10.1)	7 (8.2)	0.08
	21-29	27 (34.1)	25 (29.4)	
	30-39	29 (36.7)	35 (41.1)	
	40-49	15 (18.9)	11 (12.9)	
	50-60	0 (0)	7 (8.2)	

Only 6.1% experienced intermenstrual bleeding after vaccination. When observing the factors related to intermenstrual bleeding, there was no significant association between the incidence of bleeding, the type of booster vaccine, or the history of COVID-19 infection (p=0.98) (Table 3).

**Table 3:** Factors related to intermenstrual bleeding post COVID-19 vaccination

Variables		Intermenstrual bleeding		P-value, n=164
		Yes, 10 (6.1)	No, 154 (93.9)	
Type of vaccine received	Same type of vaccine	1 (10)	39 (25.3)	0.249
	Multiple types of vaccine	9 (90)	115 (74.6)	
Type of vaccine 1 dose	AstraZeneca	6 (60)	23 (14.9)	0.043
	Pfizer	3 (30)	70 (45.4)	
	Moderna	0	18 (11.6)	
	CanSino	0	4 (2.5)	
	Johnson	0	17 (11)	
	Sputnik and Covaxin	0	12 (7.7)	
	Sinovac	1 (10)	10 (6.4)	
	Others	0	0	
Previous COVID-19 infection	Yes	2 (20)	92 (59.7)	0.98
	No	1 (10)	35 (22.7)	
	I am not sure	7 (70)	27 (17.5)	

The most frequent menstrual alteration after vaccination was menorrhagia, an increase in the amount of bleeding in 20.7% of women. The vaccine with the highest presence of menorrhagia was AstraZeneca at 20.5%, followed by Moderna and Pfizer. And the women who reported the least amount of bleeding went with the AstraZeneca vaccine (25.5%) (Table 4).

**Table 4:** Type of vaccine and changes in menstrual flow

Vaccine dose	Type of vaccine	Type of menstrual alterations				P-value
		Without alterations, n=114 (%)	Increased bleeding, n=34 (%)	Decreased bleeding, n=16 (%)	Total, n=164	0.36
1 dose	AstraZeneca	12 (10.5)	7 (20.5)	4 (25)	23	
	Pfizer	58 (50.8)	12 (35.2)	4 (25)	74	
	Moderna	14 (12.2)	6 (17.6)	2 (12.5)	22	
	CanSino	3 (2.6)	0	1 (6.2)	4	
	Johnson	11 (9.6)	2 (5.8)	4 (25)	17	
	Sputnik	8 (7)	4 (11.7)	1 (6.2)	13	
	Sinovac	8 (7)	3 (8.8)	0	11	

The second most frequent alteration that occurred was the presence of menstrual cycles before 21 days (18.9%) and of these alterations, the Pfizer vaccine was the most common in 32.2%.

Regarding the lateration in the number of days of bleeding, the most frequent was the increase in days, bleeding more than eight days with the Pfizer vaccine in 33.3%, and women who bled less than three days with the Pfizer vaccine in 35% (Table 5).

**Table 5:** Type of vaccine and changes in cycle length

Vaccine dose	Type of vaccine		Type of menstrual alterations					P-value
		Without alterations, n=82 (%)	More than eight days of bleeding, n=21 (%)	Less than three days of bleeding, n=14 (%)	Menstrual cycles of less than 21 days, n=31 (%)	Menstrual cycles greater than 35 days, n=16 (%)	Total, n=164	0.32
1 dose	AstraZeneca	8 (9.7)	6 (28.5)	3 (21.4)	4 (12.9)	2 (12.5)	23	
	Pfizer	46 (56)	7 (33.3)	5 (35.7)	10 (32.2)	6 (37.5)	74	
	Moderna	11 (13.4)	3 (14.2)	0	4 (12.9)	4 (25)	22	
	CanSino	2 (2.4)	0	1 (7.1)	0	1 (6.2)	4	
	Johnson	7 (8.5)	2 (9.5)	4 (28.5)	4 (12.9)	0	17	
	Sputnik	6 (7.3)	1 (4.7)	0	4 (12.9)	2 (12.5)	13	
	Sinovac	2 (2.4)	2 (9.5)	1 (7.1)	5 (16.1)	1 (6.2)	11	

Among patients who presented menstrual disorders, the majority (22%) reported that their cycles were altered in two cycles and subsequently returned to normal.

Pain during menstruation occurred in 45 vaccinated patients (27.4%) and was more frequent with the Pfizer vaccine.

## Discussion

Menstrual bleeding is considered an effective measure to assess gynecological health in women. The incidence of irregular menstruation in the literature ranges from 5% to 35.6%, varying according to occupation, age, and area of residence [10,11].

During the COVID-19 pandemic, various factors, including viral infection, stress, and vaccination, contributed to changes in women's menstrual cycles. Our study found that nearly half (49%) of vaccinated women experienced altered menstrual bleeding, such as menorrhagia or metrorrhagia. Recent studies from different countries, including the United States, Norway, Hungary, and the Middle East and North Africa (MENA) region, have also reported a high incidence of menstrual changes after vaccination, ranging from 40% to 66%. However, it is worth noting that fewer than a third of the participants reported heavy menstrual flow, suggesting potential recall and selection biases in previous studies [12,13].

Our findings are consistent with the results of recent preliminary research conducted in the United States, which included 39,129 participants, where 42% reported an increase in bleeding after receiving the vaccine. Additionally, another preliminary study conducted in the United Kingdom, which retrospectively examined 4,989 premenopausal vaccinated participants, found that only 20% did not experience any menstrual abnormalities up to four months after receiving the first dose of the COVID-19 vaccine [14].

Menstrual alterations have been noted with various vaccines, including HPV, although their exact mechanism remains unclear [4]. In our study, we found no correlation between specific vaccine types and menstrual irregularities. However, women vaccinated with multiple types of vaccines showed higher rates of menstrual alterations at 36.6%. These findings align with recent studies suggesting no differences between mRNA and adenovirus-vectorized vaccines in causing such changes. It is plausible that these alterations stem from the post-vaccination immune response affecting the hypothalamic-pituitary-ovarian axis, which regulates the menstrual cycle, rather than any specific vaccine component [14].

Our research marks a milestone in examining the impact of COVID-19 vaccines on menstrual and non-menstrual bleeding in the region, regardless of the type of vaccine administered, although it has certain limitations. Being a cross-sectional study, there is a higher probability of recall biases, complicating the determination of causal relationships. Additionally, our study focused on individuals who had received up to four vaccine doses, so additional effects after subsequent doses are still unknown.

Overall, we observed that menstrual alterations following vaccination occurred in 30.5% of cases after one week and 86.8% after one month. Furthermore, 93.6% of symptoms resolved within two months. Regarding the timing of symptom onset, we found that 46.7% occurred after the first dose, 32.4% after the second dose, and 20.9% after both doses. When analyzing different vaccines, differences in the incidence of menstrual abnormalities between AstraZeneca, Sinopharm, and Pfizer were not statistically significant, with rates of 68.4%, 66.2%, and 65.4%, respectively. In our study, menstrual alterations following vaccination were much higher with Pfizer at 12.6% and Moderna at 6.4%.

In this research, women who received multidose vaccinations had a 36% higher rate of menstrual alterations compared to women with single-dose vaccinations at 11%. Our study stands out for addressing the wide diversity of participants, something that has not been frequently explored in previous research in our country. This broad sample allowed us to estimate the prevalence of menstrual abnormalities after vaccination more accurately. Additionally, we had a rigorous data collection instrument, validated by experts, which helped us to accurately and completely capture the issue at hand. Consequently, we are confident that our data accurately reflect the menstrual abnormalities experienced by women after vaccination.

Online surveys may disproportionately include or exclude certain demographics, especially those with limited internet access or technology familiarity. Nonetheless, in the current epidemiological landscape, online questionnaires remain the most efficient and secure data collection method [13].

Laganà et al. conducted a study from September 10 to October 10, 2021, with 369 responses, of which 164 were included. After the first vaccine dose, 94 participants experienced menstrual alterations, and 84 reported alterations after the second dose. Between 50% and 60% of vaccinated fertile women suffered menstrual alterations, irrespective of the vaccine type, possibly due to vaccine-induced procoagulant and proinflammatory changes [15]. Our study resembles the prevalence of menstrual alterations in the study by Laganà et al. [15], and the sample number was exactly the same.

Trogstad et al. conducted a study on menstrual disturbances post COVID-19 vaccination. They initially had 12,623 individuals aged 18-30, with 5,688 women selected. The study compared pre- and post-vaccination menstrual cycles. Heavy bleeding increased after vaccination but returned to normal around two months after the first dose, aligning with our findings in Mexico [16].

Male [17] conducted a study with 253 women planning to get vaccinated. She found a delay in menstruation after vaccine doses, but it was regulated early, with no changes in menstrual flow or other adverse effects. Alvergne et al. [14] conducted a study with 4,989 vaccinated women in the United Kingdom, showing significant menstrual disturbances, associated with factors such as oral contraceptives and past COVID-19 infection. Unlike our study, they excluded women with contraceptives or prior menstrual disorders [14-17].

## Conclusions

The incidence of menstrual disorders post COVID-19 vaccination in this study was 49%. Menstrual alterations in patients who received multiple doses and a single regimen were similar at 47% and 48%, where there is no statistical significance. The greatest number of menstrual alterations was seen in the first dose at 36%, probably due to the immunity they acquired after the different types of vaccination. Vaccination is a very effective way to prevent the severity of COVID-19 infection; it has an impact on menstrual bleeding in terms of menorrhagia and metrorrhagia. Vaccination against COVID-19 is associated with small changes in the menstrual cycle, without statistical significance. Women receiving the first dose of the vaccine had changes in the amount of bleeding specifically the amount. COVID-19 vaccination may affect menstrual bleeding, but there is no significant association with intermenstrual bleeding. Our study has limitations, such as the self-assessment of menstrual characteristics and the lack of hormone level measurements. Variations in the menstrual cycle may depend on the timing of vaccination relative to cycle phases. We must not forget that menstrual alterations post COVID-19 vaccination may result from a combination of factors, among which stress plays a crucial role. Whether chronic or acute, stress can influence the delicate hormonal balance regulating the menstrual cycle. It directly impacts the endocrine system, altering the production and release of hormones such as estrogen and progesterone, which are essential for normal menstrual cycling.
